# Activation of innate immunity during development induces unresolved dysbiotic inflammatory gut and shortens lifespan

**DOI:** 10.1242/dmm.049103

**Published:** 2021-08-27

**Authors:** Kyoko Yamashita, Ayano Oi, Hina Kosakamoto, Toshitaka Yamauchi, Hibiki Kadoguchi, Takayuki Kuraishi, Masayuki Miura, Fumiaki Obata

**Affiliations:** 1 Department of Genetics, Graduate School of Pharmaceutical Sciences, The University of Tokyo, Tokyo 113-0033, Japan; 2 Laboratory for Nutritional Biology, RIKEN Center for Biosystems Dynamics Research, Kobe 650-0047, Japan; 3 Faculty of Pharmacy, Institute of Medical, Pharmaceutical and Health Sciences, Kanazawa University, Shizenken, Kakuma-machi, Kanazawa, Ishikawa 920-1192, Japan; 4 Laboratory of Molecular Cell Biology and Development, Graduate School of Biostudies, Kyoto University, Kyoto 606-8501, Japan

**Keywords:** *Drosophila* genetics, Gut, Innate immunity, Lifespan, Microbiota

## Abstract

An early-life inflammatory response is associated with risks of age-related pathologies. How transient immune signalling activity during animal development influences life-long fitness is not well understood. Using *Drosophila* as a model, we find that activation of innate immune pathway Immune deficiency (Imd) signalling in the developing larvae increases adult starvation resistance, decreases food intake and shortens organismal lifespan. Interestingly, lifespan is shortened by Imd activation in the larval gut and fat body, whereas starvation resistance and food intake are altered by that in neurons. The adult flies that developed with Imd activation show sustained Imd activity in the gut, despite complete tissue renewal during metamorphosis. The larval Imd activation increases an immunostimulative bacterial species, *Gluconobacter* sp., in the gut microbiome, and this dysbiosis is persistent to adulthood. Removal of gut microbiota by antibiotics in the adult fly mitigates intestinal immune activation and rescues the shortened lifespan. This study demonstrates that early-life immune activation triggers long-term physiological changes, highlighted as an irreversible alteration in gut microbiota, prolonged inflammatory intestine and concomitant shortening of the organismal lifespan.

## Abstract (precis)

**Editor's choice:** This study took advantage of *Drosophila* genetics to demonstrate how mild inflammation in early life can be a risk factor of life-long health via alteration of the gut microbiota.

## INTRODUCTION

Immunity needs to be controlled tightly because both shortages and excesses of immune activation are detrimental to organisms. A chronic, and often systemic, inflammatory response occurs during ageing, which can increase the risk of various age-related diseases (Franceschi and Campisi, 2014). *Drosophila melanogaster* is a genetically tractable model for studying how immune pathways are involved in the ageing process. The Immune deficiency (Imd) pathway is an evolutionarily conserved immune regulator in *Drosophila*, which is a counterpart of the tumour necrosis factor receptor (TNFR) pathway in mammals (Buchon et al., 2014). The Imd pathway is activated upon infection with bacteria possessing DAP-type peptidoglycan and is known to be activated spontaneously in aged animals, at least in part in a gut microbiota-dependent manner (Buchon et al., 2009). Removal of microbiota or overexpression of negative regulators for the Imd pathway in the adult gut attenuates age-related Imd activation and concomitantly extends lifespan (Clark et al., 2015; Guo et al., 2014; Obata et al., 2018). Activation of the Imd pathway in gut progenitor cells induces hyperproliferation of intestinal stem cells (Petkau et al., 2017). A chronic inflammatory condition in aged flies triggers neurodegeneration and shortens lifespan, which can be rescued by inhibition of Imd signalling in glial cells (Kounatidis et al., 2017). Age-related activation of the Imd pathway in the renal (Malpighian) tubules induced by a commensal organism, *Acetobacter persici*, triggers age-dependent metabolic shifts, including purine metabolism (Yamauchi et al., 2020). These studies have revealed that age-related immune activation in various tissues leads to organismal ageing.

Early-life environmental stressors have prolonged effects on adult health, which is often described as the ‘developmental origins of health and diseases (DOHaD) hypothesis’ (Fleming et al., 2018; Hanson and Gluckman, 2014; Preston et al., 2018). In *Drosophila*, dietary protein restriction only in the larval stage extends lifespan via altered lipid metabolism (Stefana et al., 2017). Developmental exposure to low-dose oxidant remodels the gut microbiome and extends lifespan (Obata et al., 2018). Hypoxic conditions during development decrease starvation resistance and lifespan (Polan et al., 2020). These studies illustrate how environmental factors during development can program adult physiology and lifespan.

Various stressors regarded as risk factors for age-related diseases, such as malnutrition, irradiation, chemical exposures, smoke, alcohol or even mental stress, commonly lead to an inflammatory response. A longitudinal cohort study suggested that childhood infection is correlated with the incidence of cardiovascular diseases in 40-year-old humans (Burgner et al., 2015). This and many other epidemiological studies have implied that early-life inflammation is associated with inflammatory diseases and mortality in adulthood (Finch and Crimmins, 2004; Furman et al., 2019); however, few studies have tested the causal relationship directly. Irradiation during development increases cell death in the adult brain and decreases locomotive ability and organismal lifespan in *Drosophila* (Sudmeier et al., 2015a). In this state, persistent immune activation is observed in adult flies (Sudmeier et al., 2015b). By contrast, oral infection with *Erwinia carotovora* (Ecc15) in larvae does not affect the lifespan of adult flies (Houtz et al., 2019). Genetic manipulation is useful to test how early-life signalling activity impacts adult lifespan. For example, induction of mitochondrial stress triggers immune-related genes in larvae, which is associated with increased lifespan through prolonged Foxo activation (Jensen et al., 2017). Decreasing mitochondrial electron transport by knocking down *ND-75* specifically in muscles on the first day of the larval stage can extend lifespan (Owusu-Ansah et al., 2013). It is likely that infection and other stressors trigger not only immune activation but also complex reactions, such as the tissue injury/recovery response or metabolic remodelling. Thus, despite the implication that the early-life immune response affects organismal lifespan, whether an immune signalling activity during development influences the lifespan and adult physiology has not been assessed directly.

In this study, we attempt to clarify whether immune activation in a larval stage-restricted manner can alter adult fitness and lifespan. We find, in adult flies upon larval Imd activation, that immune and metabolic alteration occurs and shortens organismal lifespan.

## RESULTS

### Establishment of mild immune activation during development

**Fig. 1.**
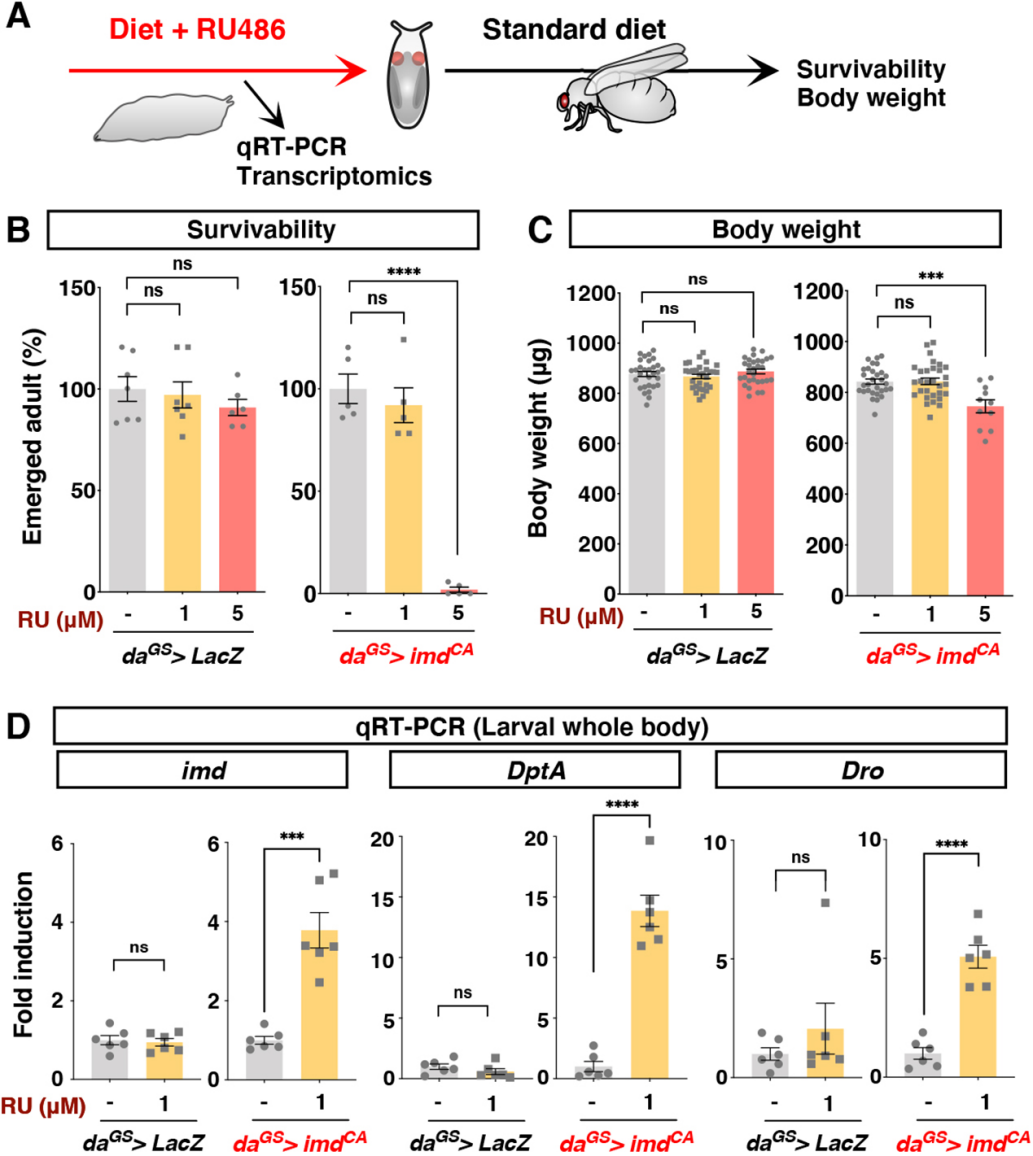
**Transient Imd activation using GeneSwitch (GS) system without strongly disturbing development.** (A) Experimental scheme. (B) Developmental survivability of flies expressing a constitutively active form of *imd* (*imd^CA^*) or negative control (*LacZ*). Average numbers of flies successfully developed in each set of conditions are shown. The *daughterless* GS driver (*da^GS^*) is used to induce gene expression ubiquitously with RU486 (RU). *n*=7 vials for *LacZ* RU^−^ and RU 1 μM; *n*=6 vials for *LacZ* RU 5 μM; *n*=5 vials for *imd^CA^*. Statistics: one-way ANOVA with Sidak's test. (C) Adult body weight. One-week-old adult male flies are used. *n*=30 individual flies, except *n*=11 for *da^GS^>imd^CA^* 5 μM RU. (D) Quantitative RT-PCR of *imd* and its target genes *Diptericin A* (*DptA*) and *Drosocin* (*Dro*) in the whole body of third-instar larvae. 1 μM RU was used to induce *imd^CA^* or *LacZ*. *n*=6. Statistics: Student's two-tailed *t*-test. ***P<0.001; ****P<0.0001; ns, not significant. All experimental results were reproduced at least twice. Samples are biological, not technical, replicates.

We used the GeneSwitch (GS) system to achieve precise control of gene manipulation (Osterwalder et al., 2001). GS is a useful tool to overexpress any gene of interest by treatment with an inducer, RU486/mifepristone, in a dose-dependent manner. For activation of the innate Imd pathway in larvae, we overexpressed a constitutive active form of *imd* (*imd^CA^*) using a ubiquitous driver, *daughterless GS* (*da^GS^*). *imd*^*CA*^ lacks the N-terminal inhibitory domain and is therefore active in the absence of bacterial stimulation (Petkau et al., 2017). We put embryos of *da^GS^>imd^CA^* and its negative control, *da^GS^>LacZ*, on top of the standard *Drosophila* diets containing various doses of RU486 and allowed them to develop into adult flies (Fig. 1A). To minimise the difference in genetic backgrounds, these flies were backcrossed eight generations. Feeding 200 μM RU486, the concentration frequently used for adult flies, caused higher developmental lethality even for the control flies in our laboratory conditions. RU486 is known to have side-effects on the physiology and lifespan of flies, depending on various factors, such as sex, mating, diet, genetic background and dose of the drug (Landis et al., 2015; Ma et al., 2021; Robles-Murguia et al., 2019; Yamada et al., 2017). Therefore, we optimised the dosage carefully by checking the control animals. Decreasing the RU486 concentration to 5 μM resulted in little effect on viability and adult body weight for the control animals (*da^GS^>LacZ*), but higher lethality and decreased body weight for *da^GS^>imd^CA^*, suggesting that strong Imd activation impairs larval growth (Fig. 1B,C). When the concentration of RU486 was decreased to 1 μM, adult *da^GS^>imd^CA^* flies showed normal body weight (Fig. 1C). At this concentration, we observed mild developmental delay compared with the control (no RU486 treatment), but this was attributable to a side effect of RU486, because the phenotype was also obvious for *da^GS^>LacZ* flies (Fig. S1A).

We first confirmed that gene expression was induced with a concentration as low as 1 μM RU486, as visualised by green fluorescent protein (GFP) expression (Fig. S1B). The driver activity is detected in the larval brain, the fat body, the gut and the Malpighian tubules (Fig. S1C; note that we used 5 μM RU486 in order to visualise the expression clearly). In the whole body of *da^GS^>imd^CA^* third-instar larvae, *imd* gene was upregulated upon treatment with 1 μM RU486 (Fig. 1D). To quantify the level of Imd activation, we performed quantitative RT-PCR analysis for antimicrobial peptide (AMP) genes regulated by the Imd signalling pathway. As expected, both Imd target genes, *Diptericin A* (*DptA*) and *Drosocin* (*Dro*), were increased only when *imd*^*CA*^ was induced by RU486 (Fig. 1D). These genes were upregulated mildly in various tissues, such as the brain, the gut and the fat body (Fig. S2A). We also performed transcriptomic profiling by 3′ mRNA-sequencing analysis using the gut tissue. AMPs predominantly regulated by the Imd pathway were all upregulated, whereas those regulated by other immune pathways were not, suggesting that Imd signalling was activated specifically in this experimental setting (Fig. S2B, Table S1). The list of differentially expressed genes did not contain typical damage-responsive genes, such as *upd3*, which is known to be increased massively in the larval gut upon oral infection with Ecc15 (Houtz et al., 2019). The induction of AMPs and Imd was suppressed in the whole body of young adult male flies (Fig. S2C). Therefore, in this experimental setting, we can increase the Imd signalling pathway specifically and mildly in a juvenile-restricted manner.

### Larval immune activation influences adult fitness

**Fig. 2.**
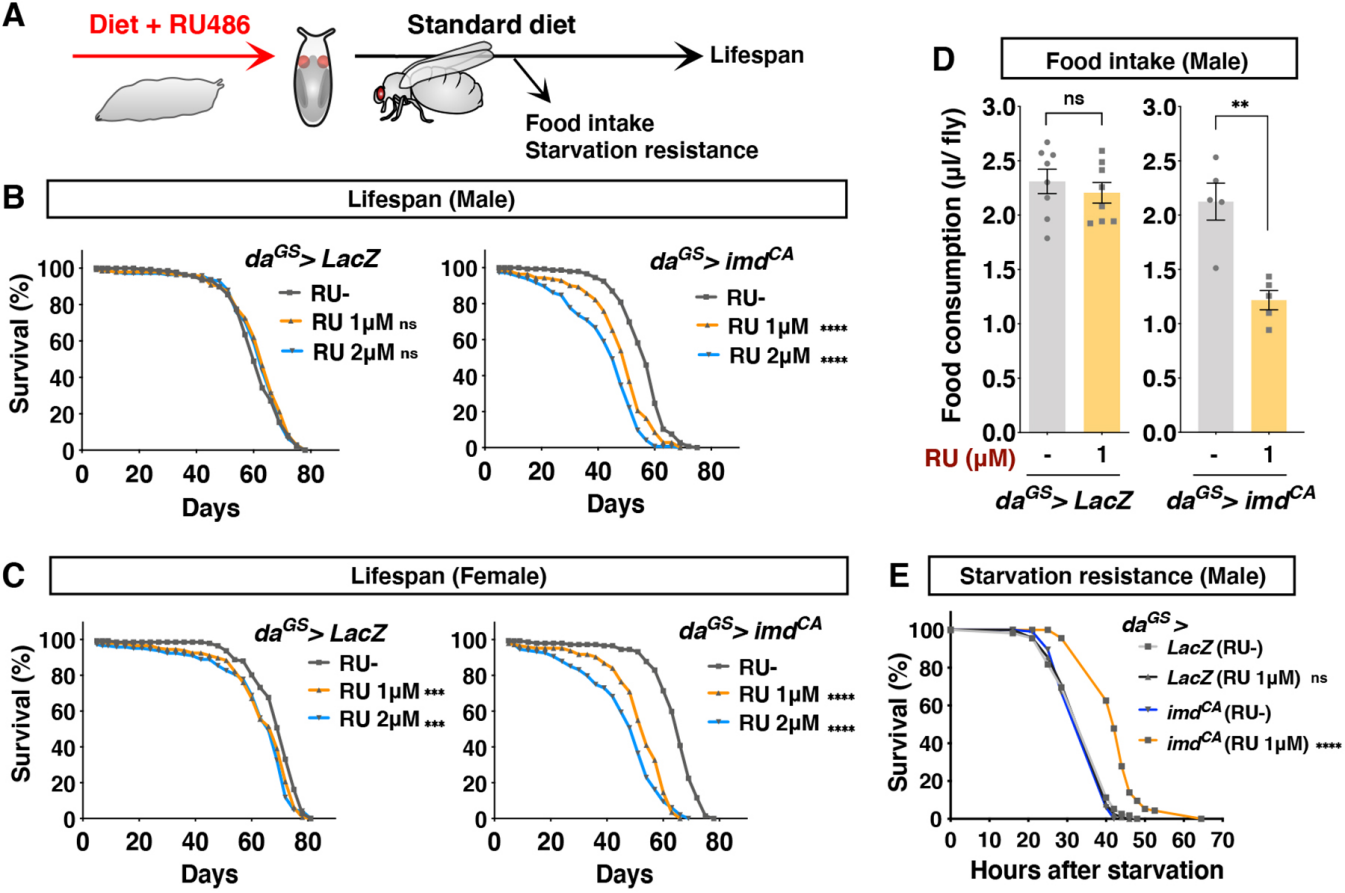
**Larval Imd activation leads to shortened lifespan, decreased food intake and increased starvation resistance in the adult.** (A) Experimental scheme. Food intake and starvation resistance were measured at 1 week after eclosion. (B,C) Lifespan of male (B) and female (C) flies with RU treatment during development. *da^GS^* was used to induce *imd^CA^* or negative control (*LacZ*) ubiquitously with 1 μM or 2 μM RU in the larval stage. For males, *n*=138 *LacZ* RU^−^, *n*=143 *LacZ* RU 1 μM, *n*=141 *LacZ* RU 2 μM, *n*=146 *imd^CA^* RU^−^, *n*=141 *imd^CA^* RU 1 μM and *n*=144 *imd^CA^* RU 2 μM. For females, *n*=142 *LacZ* RU^−^, *n*=146 *LacZ* RU 1 μM, *n*=144 *LacZ* RU 2 μM, *n*=147 *imd^CA^* RU^−^, *n*=145 *imd^CA^* RU 1 μM and *n*=148 *imd^CA^* RU 2 μM. Statistics: log-rank test. (D) Food intake of adult male flies assessed by a capillary feeder assay. 1 μM RU was used to induce *imd^CA^* or *LacZ*. *n*=8 for *LacZ* and *n*=5 for *imd^CA^*. Statistics: Student's two-tailed *t*-test. Each graph shows the mean±s.e.m. (E) Survival curve for adult male flies in starvation conditions (1% agar). 1 μM RU in the larval diet was used to induce *imd^CA^* or *LacZ*. *n*=115 *LacZ* RU^−^, *n*=117 *LacZ* RU 1 μM, *n*=115 *imd^CA^* RU^−^ and *n*=115 *imd^CA^* RU 1 μM. Statistics: log-rank test. **P<0.01, ***P<0.001; ****P<0.0001; ns, not significant.

The magnitude of Imd activation in the larvae of *da^GS^>imd^CA^* fed with 1 μM RU486 was weak enough to avert disturbance of developmental growth. We questioned whether this sublethal, transient immune activation in developing animals has a prolonged effect on adult physiology and ultimately alters lifespan (Fig. 2A). The lifespan of adult flies of *da^GS^>imd^CA^* fed with 1 μM RU486 during the larval stage was significantly shortened in both male and female flies (Fig. 2B,C). In the control (*da^GS^>LacZ*), RU486 did not affect the male lifespan at all, whereas it slightly decreased female lifespan. This side effect of RU486 on female flies, however, was not always reproducible. Given that there seemed to be no sex differences in the phenotype, we decided to use male flies mainly for the rest of the study. The lifespan shortening by larval Imd activation was dose dependent, because 2 μM RU486 decreased the lifespan further (Fig. 2B,C).

To understand how adult fitness is altered by larval Imd activation, we assessed the physiological conditions of the adult flies. In general, the amount of dietary protein was negatively correlated with lifespan. It was possible that the decrease in lifespan was attributable to the increased food intake. Unexpectedly, young male flies that had experienced larval Imd activation ate less food compared with the negative control (Fig. 2D). These data suggested that the lifespan shortening by larval Imd activation might not have been attributed to the dietary protein intake. Surprisingly, despite the decrease in food intake, they had increased starvation resistance (Fig. 2E). Larval Imd activation did not alter paraquat (oxidant) resistance, and it did induce hypersusceptibility to high salt stress (Fig. S3). These phenotypes invalidated the possibility that the shortening of lifespan by larval Imd activation was attributable simply to the increased susceptibility to stresses (general sickness of the flies). Taking these results together, we concluded that Imd activation during development induces prolonged physiological changes in adult flies and decreases lifespan. It is important to note that we could not deny the possible stress response caused by a combination of Imd activation and a side effect of RU486. However, our model might provide an accessible tool to study how larval inflammatory condition can be a risk factor for healthy adult lifespan.

### Starvation resistance and lifespan are distinctively regulated by larval Imd activation

**Fig. 3.**
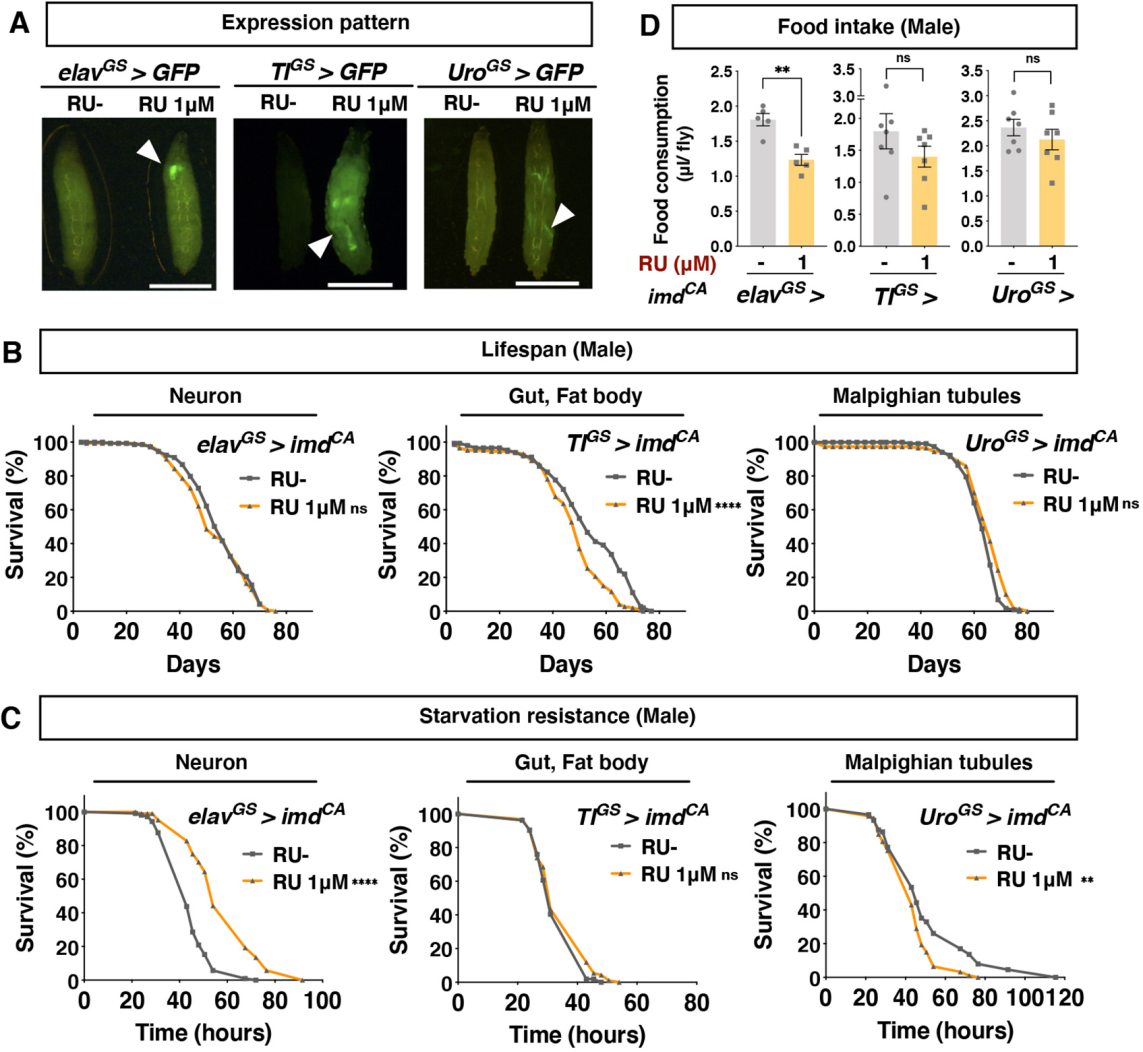
**Tissue-specific effect of larval Imd activation on adult phenotypes.** (A) Expression pattern of each GS driver upon 1 μM RU. Arrowheads indicate GFP expression. Scale bars: 1 mm. (B) Lifespan of male flies. GS drivers were used to induce *imd^CA^* with 1 μM RU in the larval stage. *n*=144 *elav^GS^* RU^−^, *n*=141 *elav^GS^* RU 1 μM, *n*=148 *TI^GS^* RU^−^, *n*=146 *TI^GS^* RU 1 μM, *n*=123 *Uro^GS^* RU^−^ and *n*=146 *Uro^GS^* RU 1 μM. (C) Survival curve for male flies in starvation conditions (1% agar). 1 μM RU was used to induce *imd^CA^*. *n*=105 *elav^GS^* RU^−^, *n*=104 *elav^GS^* RU 1 μM, *n*=104 *TI^GS^* RU^−^, *n*=92 *TI^GS^* RU 1 μM, *n*=88 *Uro^GS^* RU^−^ and *n*=93 *Uro^GS^* RU 1 μM. Statistics: log-rank test. (D) Food intake of adult male flies assessed by capillary feeder assay. *n*=5 vials for *elav^GS^* and *n*=7 vials for *Uro^GS^* and *TI^GS^*. 1 μM RU in the larval diet was used to induce *imd^CA^*. Each graph shows the mean±s.e.m. Food intake and starvation resistance were measured at 1 week after eclosion. Statistics: Student's two-tailed *t*-test. **P<0.01; ****P<0.0001; ns, not significant.

To identify which tissue(s) shortens lifespan upon Imd activation, we overexpressed *imd^CA^* in a tissue-specific manner. We used GS drivers for neurons (*elav^GS^*), the gut and the fat body (*TI^GS^*), and the Malpighian tubules (*Uro^GS^*) (Fig. 3A; Fig. S4). *TI^GS^* is often used as a gut-specific driver in the adult, but there is strong driver activity also in the fat body in the larva (Fig. S4). We observed that overexpression of *imd^CA^* only by *TI^GS^* decreased lifespan, suggesting that Imd activation in the larval gut and/or fat body induces shortened lifespan (Fig. 3B). Interestingly, however, starvation resistance was not elevated in *TI^GS^>imd^CA^* flies, but this phenotype was observed in *elav^GS^>imd^CA^* flies (Fig. 3C). Therefore, shortened lifespan and starvation resistance are distinctive phenotypes triggered by the Imd activity in the different tissues. Likewise, the decreased food intake was induced only when *elav^GS^* was used to drive Imd activation (Fig. 3D). The data indicate that food intake and starvation resistance are correlated, while the lifespan shortening occurs in parallel. In this study, we focused on the lifespan phenotype. Given that the phenotype of lifespan shortening by *TI^GS^>imd^CA^* is often weaker than that of *da^GS^>imd^CA^*, we used the *da^GS^>imd^CA^* for the rest of the study.

### Larval Imd activation causes spontaneous immune activation in the adult gut

**Fig. 4.**
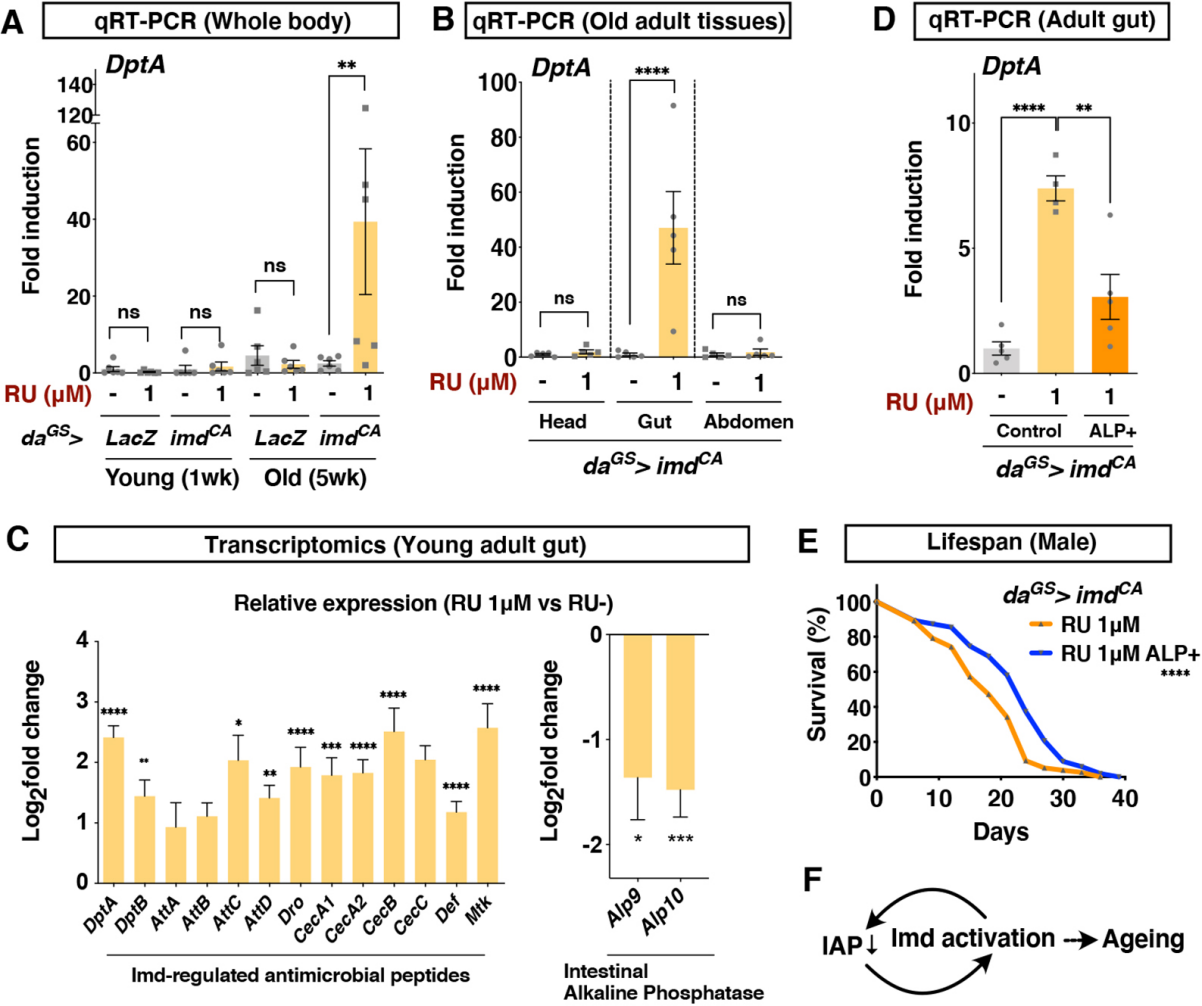
**Larval Imd activation induces inflammatory intestine in adult.** (A) Quantitative RT-PCR of Imd target gene *Diptericin A* (*DptA*) in the whole body of male flies. *da^GS^* was used to induce *imd^CA^* or negative control (*LacZ*) ubiquitously with 1 μM RU at the larval stage. *n*=6. Statistics: one-way ANOVA with Sidak's test. (B) Quantitative RT-PCR of Imd target gene *DptA* in each body part of 5-week-old male flies. *n*=5. Statistics: Student's two-tailed *t*-test. (C) Transcriptomic analysis of the adult gut from 1-week-old *da^GS^>imd^CA^* male flies, shown relative to the negative control (no RU treatment). *n*=3. Statistics: Student's two-tailed *t*-test. (D) Quantitative RT-PCR of *DptA* in the adult gut. ALP^+^, flies fed with exogenous alkaline phosphatase supplemented on top of the fly food (100 units/vial) for 3 days. 1 μM RU in the larval diet was used to induce *imd^CA^*. *n*=5 for RU^−^ and ALP^+^ and *n*=4 for RU 1 μM control. Statistics: one-way ANOVA with Sidak's test. (E) Lifespan of *da^GS^>imd^CA^* male flies with or without life-long ALP supplementation (5 units/vial). 1 μM RU in the larval diet was used to induce *imd^CA^*. *n*=101 for control and *n*=105 for ALP^+^. Statistics: log-rank test. (F) Model. Each graph shows the mean±s.e.m. *P<0.05; **P<0.01; ***P<0.001; ****P<0.0001; ns, not significant.

Imd activity is known to increase during ageing and negatively impacts organismal lifespan. To ask whether the shortened lifespan is attributable to the accelerated inflammatory response, we quantified the expression of *DptA* in the aged flies. We found that adult male flies upon larval Imd activation showed an elevation of whole-body Imd activity at 5 weeks of age (Fig. 4A). This Imd activity was derived predominantly from the gut, because we detected a sharp increase in *DptA* expression in the gut (Fig. 4B).

The fact that developmental Imd activation increased *DptA* expression in the adult gut suggested that the transient Imd activation induced an unresolved inflammatory response in the gut. To describe the condition of the tissue, we performed a transcriptomic analysis of the young adult gut from *da^GS^>imd^CA^* flies fed with RU486 during the larval stage. On day 10 in adult male flies, antimicrobial peptides regulated by the Imd pathway were already upregulated (Fig. 4C; Table S2). We noticed that genes of the Imd pathway, but not other immunity-related pathways, were already upregulated in the gut of 6-day-old adult flies (Fig. S5). Given that *DptA* was not significantly increased in the whole body of the young flies (Fig. 4A), this induction seemed mild and specific to the gut and thus not visible when analysed in the whole-body samples. Importantly, the gene *imd* was not increased in the gut of 6-day-old male flies, suggesting that the RU486-induced gene manipulation was not active anymore (Fig. S5). These data indicated that the gut Imd activation was sustained, although the *imd^CA^* induction was transient. Although we could not deny the possibility that overexpressed *imd^CA^* protein in the larval gut remained in the adult gut, it was less likely to happen, considering that the larval gut is completely degenerated and replaced by the newly generated adult gut (Bender et al., 1997; Jiang et al., 1997; Robertson, 1936).

Gut inflammation is often associated with the dysregulation of tissue homeostasis. To assess gut pathology, we tested the expression of the Upd3/JAK/STAT pathway. Neither *upd3* nor *Socs36E*, a target of STAT transcription factor, was upregulated by larval Imd activation in gut samples from young or old flies (Fig. S6A,B). In addition, we did not observe an increase in the number of proliferating intestinal stem cells, a hallmark of tissue ageing, as shown by phospho-Histone H3 staining of the adult gut (Fig. S6C). However, we could not completely deny the possibility that there might be a difference at a later stage.

We assumed that an experience of larval immune activation augments immunity as an adaptive response to prepare for future infection in the adult flies. Given that the increased *DptA* expression is restricted to the gut, we asked whether the flies were resistant to oral infection that could be influenced by AMPs (Liehl et al., 2006). Unexpectedly, the larval Imd induction was not beneficial for adult flies against *Pseudomonas entomophila* infection; on the contrary, it increased susceptibility (Fig. S7). Therefore, persistent Imd activity in the gut might not be a protective adaptation of the organism.

### Larval Imd activation decreases anti-inflammatory intestinal alkaline phosphatase

We also noticed that intestinal alkaline phosphatases (IAPs) *Alp9* and *Alp10* were decreased in the adult gut (Fig. 4C; Table S2). Among 18 downregulated genes (fold change<0.5, *P*<0.05), two IAPs were listed in the third and fourth place, the expression of which decreased by one-third of the control. IAP is an evolutionarily conserved regulator of gut homeostasis, the expression of which is known to be downregulated during ageing (Kühn et al., 2020). IAP is a luminal protein known to be anti-inflammatory by various mechanisms, such as dephosphorylation of lipopolysaccharide or nucleotides to attenuate its immunostimulative capacity. It is reported that feeding exogenous alkaline phosphatase (ALP) can augment the gut homeostasis and extend mouse and fly lifespans (Kühn et al., 2020). Decreased IAP expression is also reported in rodents and in human patients with inflammatory bowel disease (Tuin et al., 2009). Increased IAP expression is beneficial to suppress dextran sulfate sodium-induced colitis in mice (Campbell et al., 2010). In our model, feeding adult flies that experienced larval Imd activation with a high dose of ALP (100 units/vial) suppressed *DptA* upregulation in the young adult gut (Fig. 4D). Sustained ALP (5 units/vial) supplementation throughout adult life can increase the lifespan of male flies (Fig. 4E). These data suggest that decreased IAP expression contributes to the inflammatory response in the gut and the concomitant shortened lifespan.

Decreased IAP expression did not occur in larvae upon Imd activation (Table S1). Intriguingly, *Alp10* was listed in the upregulated genes in the larval gut. Genetic activation of the Imd pathway in the adult gut decreased IAP expression in both males and females, suggesting that decreased IAP is likely to be attributable to the upregulated Imd signalling (and/or concomitant tissue senescence) in the adult (Fig. S8). This is also supported by the fact that both IAPs were decreased in enterocytes upon *P. entomophila* infection shown in the public database (http://flygutseq.buchonlab.com/). Considering that IAP can suppress Imd activity, developmental Imd activation could trigger the inflammatory vicious cycle of IAP downregulation and Imd upregulation (Fig. 4F).

### Gut microbiota exacerbates gut immune activation and shortened lifespan

**Fig. 5.**
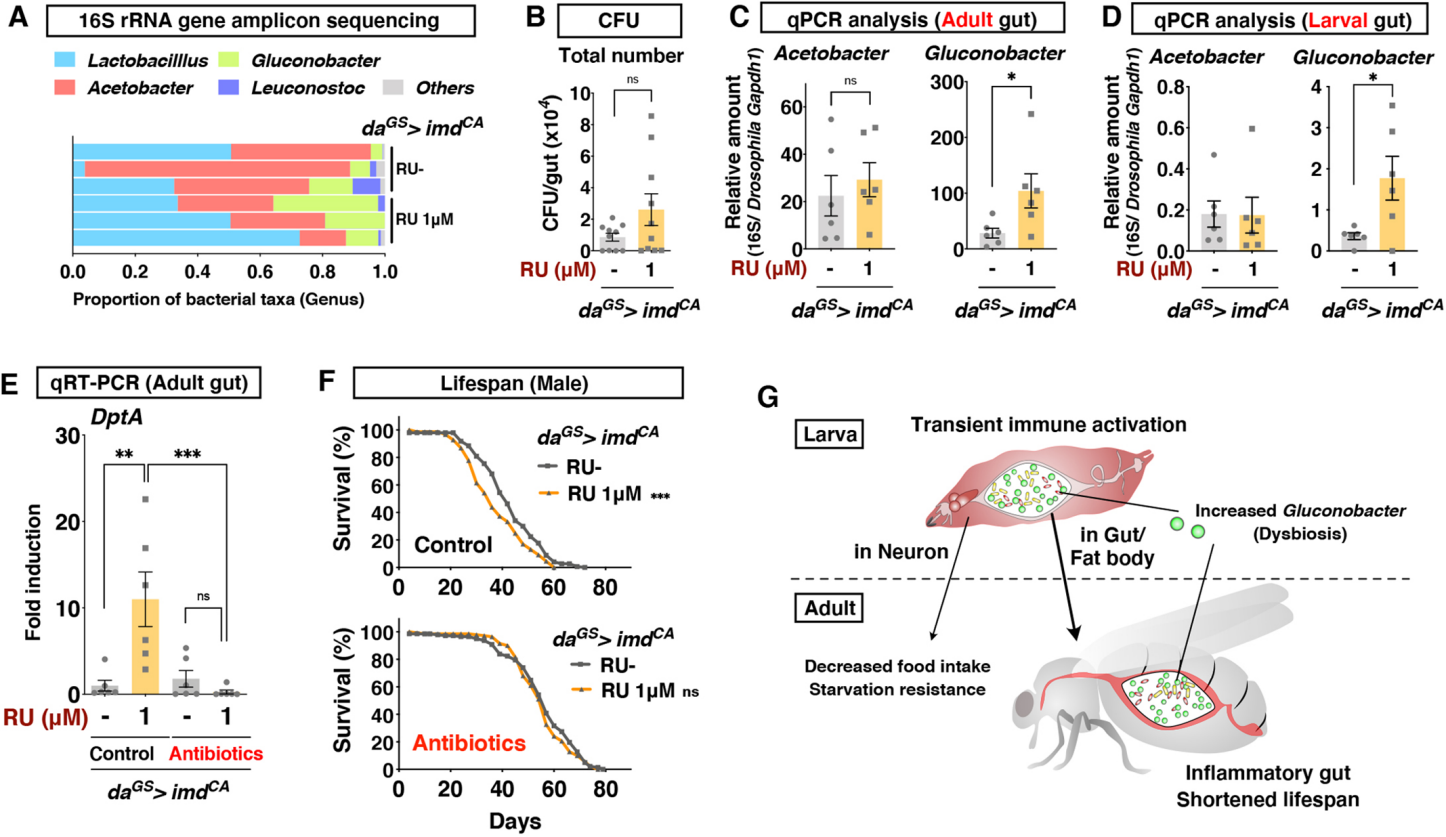
**Removal of gut microbiota rescues shortened lifespan.** (A) 16S rRNA gene amplicon sequencing analysis of 1-week-old adult male gut. Each genus is shown in a different colour. There were three biological replicates for each condition. *da^GS^* was used to induce *imd^CA^* ubiquitously with 1 μM RU in the larval stage. (B) Colony-forming unit (CFU) assay to count the number of live bacteria from the 1-week-old adult male gut. 1 μM RU was used to induce *imd^CA^*. *n*=10. Statistics: Student's two-tailed *t*-test. (C,D) Quantitative PCR of *Acetobacter* or *Gluconobacter* in 1-week-old adult male (C) or third-instar larval (D) gut. The amount is shown relative to the *Drosophila Gapdh1* gene. 1 μM RU was used to induce *imd^CA^*. *n*=6. Statistics: Student's two-tailed *t*-test. (E) Quantitative RT-PCR of Imd target gene *DptA* in 1-week-old adult male gut. 1 μM RU was used to induce *imd^CA^*. See the Materials and Methods section for the composition of the antibiotic cocktail. *n*=6. Statistics: one-way ANOVA with Sidak's test. (F) Lifespan of male flies with or without antibiotic treatment in the adult. 1 μM RU was used to induce *imd^CA^*. *n*=147 Control RU^−^, *n*=153 Control RU 1 μM, *n*=142 Antibiotics RU^−^ and *n*=141 Antibiotics RU 1 μM. Statistics: log-rank test. (G) Model. Each graph shows the mean±s.e.m. *P<0.05; **P<0.01; ***P<0.001; ns, not significant.

The gut microbiome increases innate immune activation during ageing, which shortens lifespan (Buchon et al., 2009; Clark et al., 2015). Acetobacteraceae, such as *Acetobacter aceti*, are known to increase Imd activity in aged flies, and removing them results in extended lifespan (Obata et al., 2018). Another genus of Acetobacteraceae, *Gluconobacter* spp., are known to expand in the gut microbiota in response to the host immune activation and to induce mortality of flies (Kosakamoto et al., 2020; Ryu et al., 2008). We assumed that altered gut microbiota contribute to the lifespan shortening in adult flies that experienced larval Imd activation. To test this hypothesis, we performed 16S ribosomal RNA (rRNA) gene amplicon sequencing analysis of the gut microbiota in the young adult gut. The result did not delineate a huge difference in the microbial composition (Fig. 5A). The total number of live bacteria assessed by colony-forming unit assay was also not significantly changed (Fig. 5B). However, when we quantified the number of bacteria by quantitative PCR using a set of primers detecting genera *Acetobacter* or *Gluconobacter*, we noticed that *Gluconobacter* was significantly increased (Fig. 5C). Interestingly, this alteration in gut microbiota is already present in the larval gut upon mild Imd activation (Fig. 5D). Absence of the phenotype denied the possible side effect of RU486 feeding on the gut microbiota (Fig. S9). These data suggested that the persistent increase in this immunostimulative *Gluconobacter* might mediate the prolonged effect of larval Imd activation. Although we cannot distinguish whether this dysbiosis might be a consequence or a cause of the immune response in the adult gut, the irreversible change in the gut microbiome provides another line of evidence that the flies suffer from intestinal inflammation.

To test directly whether the gut microbiota is involved in the gut inflammatory response and eventually shortens the lifespan, we fed adult flies that experienced larval Imd activation with antibiotics (rifampicin, tetracycline and ampicillin) to eliminate gut microbiota. We found that Imd upregulation in the adult gut was abolished by the antibiotic treatment (Fig. 5E). In this state, larval Imd activation did not shorten lifespan (Fig. 5F). These data suggest that gut microbiota in the adult contributed to the pathological phenotypes. Together, larval Imd activation would promote the prolonged intestinal inflammation and shortened lifespan, in part via persistently altered gut microbiota (Fig. 5G).

## DISCUSSION

In this study, we developed a model to study how developmental immune activation influences adult fitness and, ultimately, organismal lifespan. Strong activation of the immune signalling pathway inhibits developmental processes and causes lethality (DiAngelo et al., 2009; Georgel et al., 2001). By using the GS system, we set up low-grade Imd activation restricted to juveniles, which enabled us to obtain superficially normal, healthy adult flies. Nonetheless, this larval Imd activation led to the development of an adult gut with induced antimicrobial peptide genes, decreased *Alp9/10* expression and increased *Gluconobacter* spp. in the gut microbiota, all of which are hallmarks of intestinal inflammation. The increase in *Gluconobacter* is evident in the larval gut, whereas the decrease in *Alp9/10* expression occurs only in the adult stage. Therefore, it is possible that larval Imd activation triggers a gut dysbiosis-driven prolonged inflammatory response. Previous studies have reported that *Gluconobacter* was increased in response to the host immune activation and shortened *Drosophila* lifespan (Kosakamoto et al., 2020; Ryu et al., 2008). Both the gut Imd pathway and the fat body (systemic) Toll pathway can increase *Gluconobacter* in the gut (Kosakamoto et al., 2020). Increased *Gluconobacter*, in turn, stimulates the host Imd pathway, triggering a positive feedback loop between *Gluconobacter* expansion and Imd activation, at least in the presence of inflammatory cell death (Kosakamoto et al., 2020). This inflammatory vicious cycle might explain the adult pathophysiology triggered by the larval Imd activation. However, we do not have the direct evidence to link the larva-specific Imd activation functionally with the concomitant shortening of lifespan by this specific bacterium. Thus, a careful, stage-restricted gnotobiotic experiment is required to test whether the increase in *Gluconobacter* is a cause or a consequence of the intestinal inflammation and the shortened lifespan.

Another possibility is that the transient Imd activation in the larval gut simply sensitises the adult gut to inflammatory stimuli. This phenomenon might be analogous to trained immunity or innate immune memory, whereby greater protection against reinfection is achieved (Kurtz, 2005; Netea et al., 2020). We did not observe, however, any protection of adult flies that experienced larval Imd activation to oral infection despite them having increased antimicrobial peptides. This might suggest that the chronic immune activation in the adult gut is pathological rather than protective during infectious damage. Mechanistically, the sensitivity of Imd signalling can become high, owing, in part, to the dampened expression of a negative regulator of Imd, such as IAPs. We previously observed that the systemic inflammatory response in necrosis-induced flies decreases S-adenosylmethionine (SAM), a methyl donor required for histone methylation (Obata et al., 2014). In worms, SAM is decreased during infection, and this leads to decreased H3K4me3 to regulate the immune response (Ding et al., 2015). A spontaneous immune response in the aged fat body in *Drosophila* is attributed to declining Lamin C expression and epigenetic deregulation (Chen et al., 2014). Therefore, the immune-epigenetic crosstalk in the adult midgut progenitors in the larval gut would, consequently, alter the epigenetic homeostasis of the adult intestinal stem cells and/or their progeny enterocytes. The detailed mechanism of the Imd sensitisation needs to be investigated further.

It is widely accepted that early-life exposures to microorganisms, such as healthy gut microbiota, are essential for shaping appropriate immune and metabolic homeostasis (Blanton et al., 2016; Cox and Blaser, 2015; Gensollen et al., 2016; Russell et al., 2012). Inappropriate microbial exposures therefore impact various inflammation-associated diseases in later life, including inflammatory bowel disease (Hviid et al., 2011; Stiemsma and Michels, 2018). Mice born to germ-free mothers become susceptible to a high-fat diet inducing obesity, owing to the loss of immune, endocrinal homeostasis developed in the absence of bacterial metabolites (Kimura et al., 2020). Early-life disturbance of microbial composition by transient antibiotic treatment can cause obesity in adults (Cox et al., 2014; Nobel et al., 2015). Antibiotic treatment during development can also induce long-term changes in cytokine production in the brain and associated behavioural alteration (Leclercq et al., 2017). Whether immune signalling in the developmental stage provokes inflammatory diseases and affects organismal lifespan in mammals, as we observed in flies, needs to be tested.

The important question raised by the present study is how neuronal immune activation in the larval stage leads to starvation resistance and decreased food intake in adults. Infection by a pathogen decreases food intake, at least in larvae (Liehl et al., 2006). Direct Imd activation by circulating peptidoglycan in octopaminergic neurons alters oviposition but does not affect food intake (Kurz et al., 2017). As far as we know, it has not been elucidated whether activation of Imd signalling in some neurons regulates food intake in adult flies. Increased starvation resistance suggests a decrease in energy expenditure and/or augmented metabolic efficiency of the animals. An immune-metabolic switch is essential for the allocation of nutrients from anabolism to immune effector production (Clark et al., 2013). Persistent activation of the Imd pathway in the fat body leads to altered metabolism (Davoodi et al., 2019). Imd activation by gut microbiota can also modulate the metabolic homeostasis of the host (Broderick et al., 2014; Combe et al., 2014). Imd activation in enteroendocrine cells in the gut alters metabolism and development through an endocrine peptide, Tachykinin (Kamareddine et al., 2018). In our model, we assume that experiencing Imd activation triggers a physiological and metabolic adaptation in the animals to be prepared for a future infection/stress response. This long-term immunometabolic interaction is an interesting direction to be explored in a future mechanistic study.

## MATERIALS AND METHODS

### *Drosophila* stocks and husbandry

Flies were reared on a standard diet containing 4% cornmeal, 6% baker's yeast (Saf Yeast), 6% glucose (Wako, 042-31177) and 0.8% agar (Kishida Chemical, 260-01,705) with 0.3% propionic acid (Tokyo Chemical Industry, P0500) and 0.05% nipagin (Wako, 132-02635). Flies were reared at 25°C, 65% humidity with 12 h/12 h light/dark cycles. The fly lines were as follows: *da-GeneSwitch* (Tricoire et al., 2009), *UAS-lacZ* (gift from Dr Corey S. Goodman, University of California, Berkeley, CA, USA), *UAS-imd^CA^* (Petkau et al., 2017), *elav-GeneSwitch* (Osterwalder et al., 2001), *TI-GeneSwitch* (gift from Dr Scott Pletcher, University of Michigan, Ann Arbor, MI, USA), *Uro-GeneSwitch* (present study) and *UAS- 2×EGFP* (Bloomington *Drosophila* Stock Center, 6874). *da-GeneSwitch*, *UAS-lacZ* and *UAS-imd^CA^* were backcrossed eight generations with *w^iso31^*. Embryos were collected using a cage containing young (∼1-week-old) parents of GS and UAS lines and an acetic acid agar plate [2.3% agar (Becton, Dickinson and Company, 214010), 10% sucrose (Wako, 196-00015) and 0.35% acetic acid (Wako, 017-0256)] with live yeast paste. Equal volumes of the collected embryos were put onto the top of fly food containing RU486 (Tokyo Chemical Industry, M1732; dissolved in ethanol) or ethanol (as a negative control), in order to control the larval density. Adult flies eclosed within 2 days were collected and maintained for an additional 2 days for maturation on standard fly diet. Then, the flies were sorted by sex, put into vials (with 15 flies per vial) and flipped to fresh vials every 3 days.

We would like to note that the magnitude of adult phenotypes induced by larval Imd activation is variable, empirically depending on seasons. In Japan, we have typical seasonal changes in the temperature and humidity that greatly influence gut microbial composition, even though we use incubators with constant temperature/humidity settings. Therefore, we prepared a large number of flies pooled from the same batch of healthy parents and carefully compared the phenotypes of flies with or without the drug administration.

### Measurement of developmental speed, survivability and body weight

After putting an equal volume of embryos into each vial, the number of pupae at each time point was counted to assess developmental speed. Data were normalised by the total number of pupae. For survivability, the number of adult flies in each bottle was counted and divided by the number of control flies (without RU486 treatment). We included both sexes in this experiment because there was no sex bias in the survival rate. The body weight of eclosed adult flies was measured individually with an ultra-microbalance (Mettler Toledo, XPR2). For the body weight measurement, we used male flies because female body weight is greatly affected by the number of eggs inside the body, making it difficult to assess body size (i.e. larval growth).

### Quantitative RT-PCR analysis

Total RNA was purified from five male flies or three to five guts using a Promega ReliaPrep RNA Tissue Miniprep kit (z6112). The crop and the Malpighian tubules were carefully removed manually. Complementary DNA (cDNA) was made from 200 ng or 400 ng DNase-treated total RNA by a Takara PrimeScript RT Reagent Kit with gDNA Eraser (RR047B). Quantitative PCR was performed using TB Green Premix Ex Taq (Tli RNaseH Plus) (Takara Bio, RR820W) and a Quantstudio6 Flex Real Time PCR system (Thermo Fisher Scientific) or qTower3 (Analytik Jena) using *pol2* RNA as an internal control. Quantification was done with the copy number method using serial dilution of standard amplicons. Primer sequences are listed in Table S3.

### RNA-sequencing analysis for transcriptomics

Dissected larval or adult guts were homogenised in 150 μl QIAzol Lysis Reagent (Qiagen, 79306) and stored at −80°C. Triplicate samples were prepared for each experimental group, containing three to five male guts per sample. The crop and the Malpighian tubules were carefully removed manually. Then, 350 μl QIAzol Lysis Reagent was added and left for 30 min at room temperature. Chloroform (100 μl) was added and mixed by vortexing, then left for 2 min at room temperature. Using an RNeasy Plus Micro Lit (Qiagen, 74034), RNA was extracted based on the manufacturer's protocol. The RNA was sent to Kazusa Genome Technologies to perform 3′ RNA-sequencing analysis. A cDNA library was prepared using the QuantSeq 3′ mRNA-Seq Library Prep Kit for Illumina (FWD) (Lexogen, 015.384). Sequencing was done using Illumina NextSeq 500 and NextSeq 500/550 High Output Kit v.2.5 (75 cycles) (Illumina, 20024906). Raw reads were analysed by BlueBee Platform (Lexogen), which performs trimming, alignment to the *Drosophila* genome and counting of the reads. The count data were analysed statistically by Wald's test using DESeq2. The results have been deposited in DDBJ under the accession number DRA011490.

### Survival assays

For lifespan analysis, the number of dead flies was counted every 3 days. To minimise the variation between culturing vials, we used eight to 12 vials with 15 flies per vial. For high salt stress, flies were placed onto food containing 500 mM NaCl (Wako, 191-01665), 5% sucrose (Wako, 196-00015) and 1% agar (Kishida Chemical, 260-01705). For starvation stress, flies were placed in vials containing 1% agar. For oxidative stress, flies were placed onto food containing 10 mM paraquat (1,1′-dimethyl-4,4′-bipyridinium dichloride; Tokyo Chemical Industry, D3685), 5% sucrose and 1% agar. In each assay, 15 male flies per vial were incubated at 25°C, and the number of dead flies was counted several times during each day.

*Pseudomonas entomophila* wild-type strain L48 was kindly provided by Dr B. Lemaitre (École Polytechnique Fédérale de Lausanne, Lausanne, Switzerland). Oral infection was performed as described previously (Kuraishi et al., 2011). Briefly, *P. entomophila* was grown in Luria-Bertani (LB) medium at 29°C overnight and collected by centrifugation. Adult flies were incubated for 2 h at 29°C in an empty vial for starvation and then placed in a fly vial with a bacterial solution. The bacterial solution was obtained by mixing a pellet of bacteria with a culture supernatant (1:1), added to a filter paper disc that completely covered the surface of the standard fly medium. Flies were maintained at 29°C, and mortality was monitored.

### Capillary feeder assay for food intake

Two glass capillaries containing 5% sucrose, 2 mg/ml red dye (Acid Red 52, Wako, 3520-42-1) and n-octyl acetate (1:100,000; TCI, 112-14-1) were inserted into the cap. Ten male flies were placed in each vial containing 1% agar to avoid desiccation stress. The level of the food was marked, and the vials were laid in a container with wet towels to prevent water evaporation. The container was incubated at 25°C. After 24 h, the amount of food that remained in the capillaries was recorded. The vial without flies was also included in the container to subtract evaporation.

### Construction of *Uro-GeneSwitch* fly

To generate the *Uro-GeneSwitch* driver, the putative 881 bp promotor sequence of *Urate oxidase* (*Uro*) gene was amplified by PCR using *w^Dah^* genomic DNA. The sequence of GeneSwitch was amplified by PCR using pelav-GeneSwitch (Addgene, 83957). The backbone vector pelav-GeneSwitch was digested with KpnI, and ligated with the amplicons Uro promoter and GS using the NEBuilder HiFi DNA Assembly Kit (New England BioLabs, E2621X). Transgenic lines were generated using standard methods for P-element-mediated germline transformation (BestGene).

### Proliferation of intestinal stem cells

Female guts were dissected in PBS and fixed in 4% paraformaldehyde for 1 h. After washing with PBST (0.1% Triton X-100), the guts were incubated with blocking buffer (PBST with 5% normal donkey serum) for 30 min. The guts were incubated overnight at 4°C with anti-Histone H3 (phosphor S28) (rat, Abcam, ab10543) diluted 1:2000 in blocking buffer. After washing with PBST, guts were incubated for 2 h at room temperature with anti-rat IgG, 488 (Thermo Fisher Scientific, A-21208) diluted 1:1000 and Hoechst 33342 (Thermo Fisher Scientific, H3570) in blocking buffer. The number of phospho-Histone H3^+^ cells in the gut was counted manually under a fluorescent microscope.

### 16S rRNA gene amplicon sequencing analysis and quantitative PCR of bacteria

Adults were rinsed briefly in PBST, 50% (v/v) bleach (Oyalox), 70% ethanol and PBS before dissection. Male guts (six to eight per sample) without the tracheae, Malpighian tubules and crop were dissected from day 10 adult flies. Dissected guts were collected in PBS on ice, then homogenised in 270 μl lysis buffer (20 mM Tris-HCl, pH 8.0, 2 mM EDTA and 1% Triton X-100) with 20 mg/ml lysozyme from chicken egg (Sigma-Aldrich, L4919) using a tissue grinder (BMS, BC-G10) with a pestle (BMS, BC-PES50S). The homogenates were incubated at 37°C for 45 min in a 1.5 ml microcentrifuge tube, then homogenised further in a 2 ml tube (Yasui Kikai, ST-0250F-O) containing 0.1 mm glass beads (Yasui Kikai, YZB01) using a Multi-beads shocker (Yasui Kikai) at 2500 rpm for 20 s two times. To remove bubbles, the tube was centrifuged briefly. After an additional 15 min incubation at 37°C, 30 μl proteinase K and 200 μl Buffer TL (Qiagen) were added to each sample. The samples were incubated at 56°C for 15 min. Genomic DNA was purified by a QIAamp DNA Micro kit (Qiagen, 56304) and sent to Macrogen Japan, where 16S rRNA amplicon sequencing (Illumina MiSeq) and the bioinformatics analysis were performed. 16S rRNAs were amplified using primers targeting the V3 and V4 regions. The results have been deposited in DDBJ under the accession number DRA011489.

For quantification of bacterial species by quantitative PCR, three different primer sets were used for *Acetobacter* (Fridmann-Sirkis et al., 2014), *Gluconobacter* (Torija et al., 2010) and *Drosophila Gapdh1* gene for normalisation. Primer sequences are listed in Table S3. For *Acetobacter*, TB Green Premix Ex Taq (Tli RNaseH Plus) (Takara Bio, RR820W) was used. For the analysis of *Gluconobacter*, probe-based quantitative PCR was performed using PrimeTime Gene Expression Master Mix (Integrated DNA Technologies, 1055772).

### Colony-forming unit assay

One fly from each vial was surface sterilised by serial washes with 3% bleach and 70% ethanol. The gut was dissected in PBS and homogenised in PBS. Serial dilutions of the homogenate were plated by EDDY JET2 (iUL, PL0300), and the number of colonies on an MRS agar plate (Kanto Chemical, 711361-5) was counted manually after incubation for 2 days at 30°C.

### IAP supplementation

First, 5000 units/ml Quick CIP (New England BioLabs, M0525L) was diluted with enzyme buffer (25 mM Tris-HCl, 1 mM MgCl_2_, 0.1 mM ZnCl_2_ and 50% glycerol, pH 7.5, 25°C). Then, 20 μl of the CIP solution was applied directly on top of the standard diet. The enzyme buffer was used as the negative control. For quantification of *DptA* expression, the adult flies were fed with 100 units/vial for 3 days. For lifespan analysis, only 5 units/vial was used for reasons of economy to treat adult flies throughout life.

### Antibiotic supplementation

Antibiotics (200 μg/ml rifampicin, 50 μg/ml tetracycline, 500 μg/ml ampicillin, together with 0.12% nipagin) were added to the standard diet to remove all bacteria. For quantitative RT-PCR, we fed young adult male flies for 3 days. For lifespan, we fed male flies throughout adult life.

### Quantification and statistical analysis

Statistical analysis was performed using GraphPad Prism v.8 except for survival curves, for which OASIS2 was used (Han et al., 2016). The log-rank test was used to compare two survival curves. Student's two-tailed *t*-test was used to compare two samples. One-way ANOVA with Sidak's test was used to compare any combination of interest within a group. *P*<0.05 was considered statistically significant. Bar graphs were drawn as the mean±s.e.m., with all the data points shown by dots to allow readers to see the number of samples and all raw data. All experimental results were reproduced at least twice.

## Supplementary Material

Supplementary information
