# Supplementary material for: Activation of innate immunity during development induces unresolved dysbiotic inflammatory gut and shortens lifespan
Source: Dis Model Mech. 2021 Aug 27;14(9):dmm049103. doi: 10.1242/dmm.049103 (PMC8405880; doi:10.1242/dmm.049103)
Supplement: Supplementary information [file dmm-14-049103-s1.pdf]

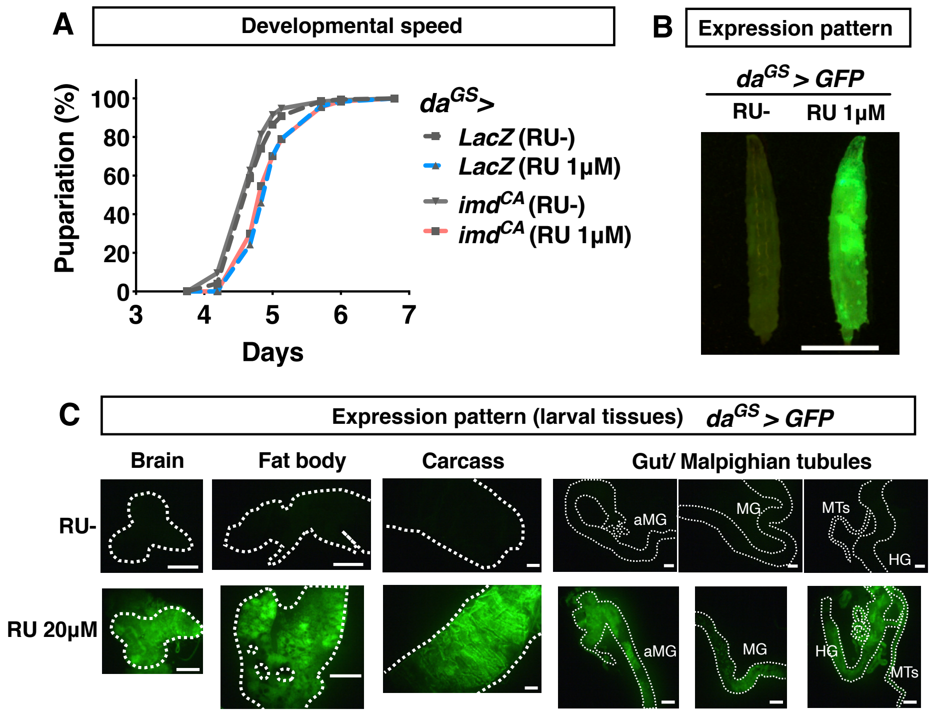

Fig. S1. Mild Imd activation does not impact developmental timing.

(A), Developmental timing of flies expressing constitutive active form of Imd (*imd*^{CA}) or negative control *(LacZ)* by *daughterless* Gene Switch driver (*da*^{GS}) with or without 1 µM RU486. n=551 LacZ RU-, n=600 LacZ RU 1 µM, n=514 *imd*^{CA} RU-, n=580 *imd*^{CA} RU 1 µM. (B), Expression pattern of *daughterless* Gene Switch driver upon 1 µM RU486 visualised by GFP. Scale bar: 1mm. (C), Expression pattern of *daughterless* Gene Switch driver in the larval tissues. To clearly visualise the driver activity, GFP was induced by 20 µM of RU486. Scale bars: 200 µm.

Disease Models & Mechanisms • Supplementary information

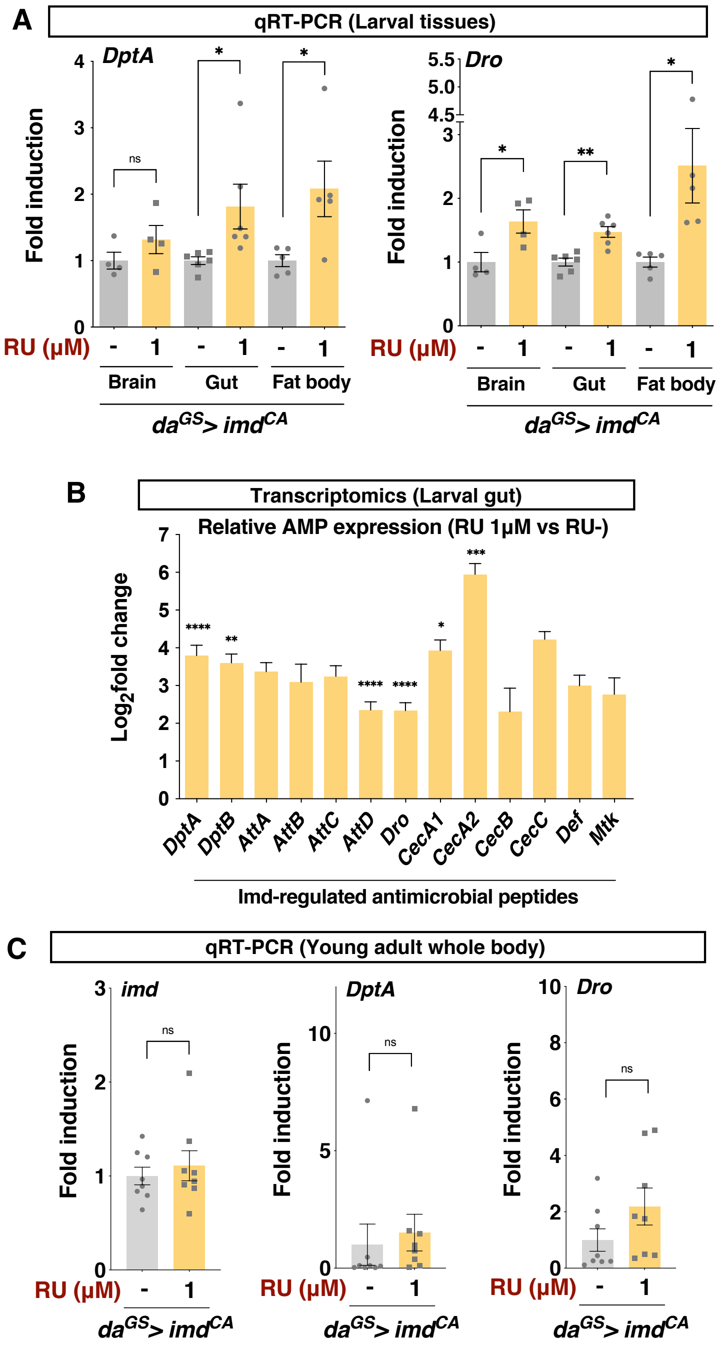

Fig. S2. Transient Imd activation in larva by the Gene Switch system.

(A), Quantitative RT-PCR of Imd target genes *Diptericin A* (*DptA*) and *Drosocin* (*Dro*) in the third instar larval organs. *daughterless* Gene Switch driver (*da*^{GS}) was used to induce constitutive active form of Imd (*imd*^{CA}) ubiquitously by 1 µM RU486. n=4 Brain, n=6 Gut, and n=5 Fat body. (B), Transcriptomic analysis of the larval gut from *da*^{GS}>*imd*^{CA} third instar larvae. Relative to the negative control (no RU486 treatment) is shown. n=3. (C), Quantitative RT-PCR of *imd* and its target genes *Diptericin A* (*DptA*) and *Drosocin* (*Dro*) in the whole body of day0 male flies. n=8. Each graph shows the mean ± SEM. Statistics: two-tailed Student's *t*-test, *p<0.05; **p<0.01; ***p<0.001; ****p<0.0001; ns, not significant.

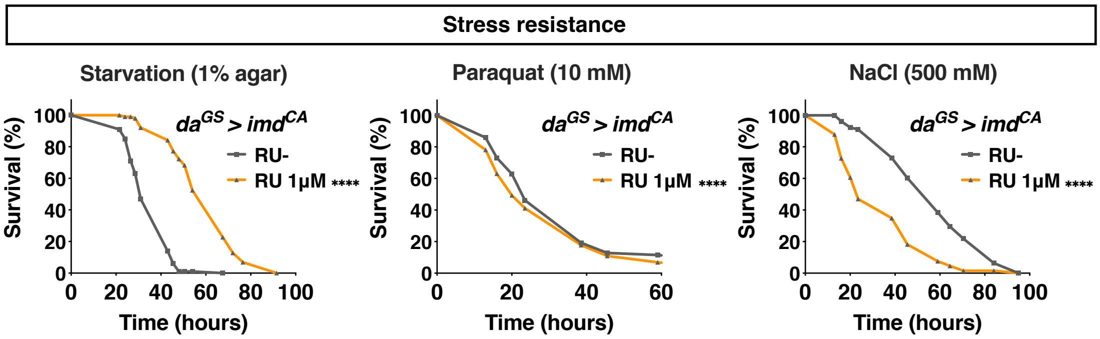

Fig. S3. Larval Imd activation alters adult resistance to stressors.

Survival curves under starvation (1% agar), paraquat (10 mM), or high-salt (NaCl 500 mM) conditions of one-week-old male flies that experienced larval Imd activation. *daughterless* Gene Switch driver (*da*^{GS}) was used to induce constitutive active form of Imd (*imd*^{CA}) ubiquitously by 1 μM RU486 in the larval stage. n=101 Starvation, n=73 Paraquat, n=66 NaCl. Statistics: log-rank test, ****p<0.0001

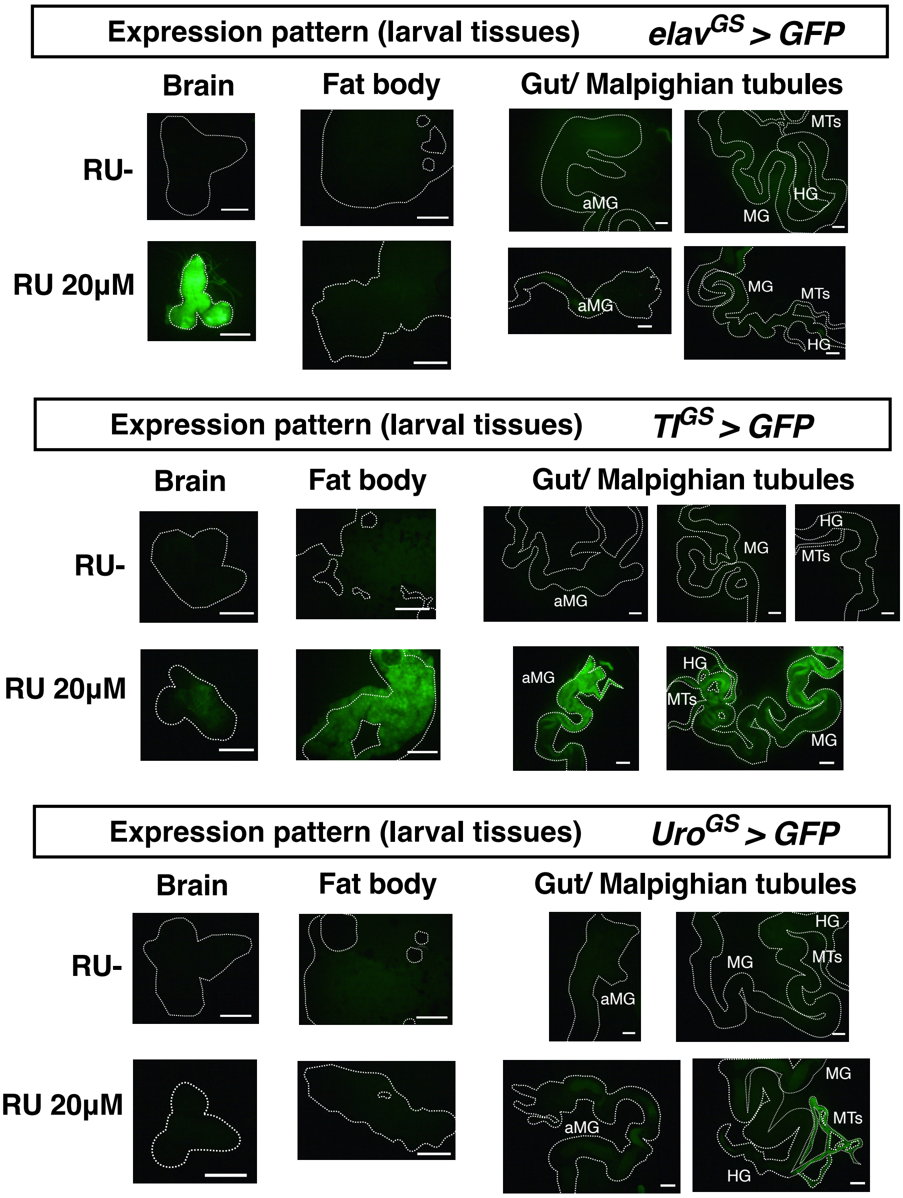

Fig. S4. Expression patterns of Gene Switch drivers in the larval tissues. GFP was expressed by *elav*^{GS}, *TI*^{GS}, and *Uro*^{GS} drivers using 20 µM RU486. Third-instar larvae were dissected and GFP was visualised by fluorescent microscopy. Scale bars: 200 µm.

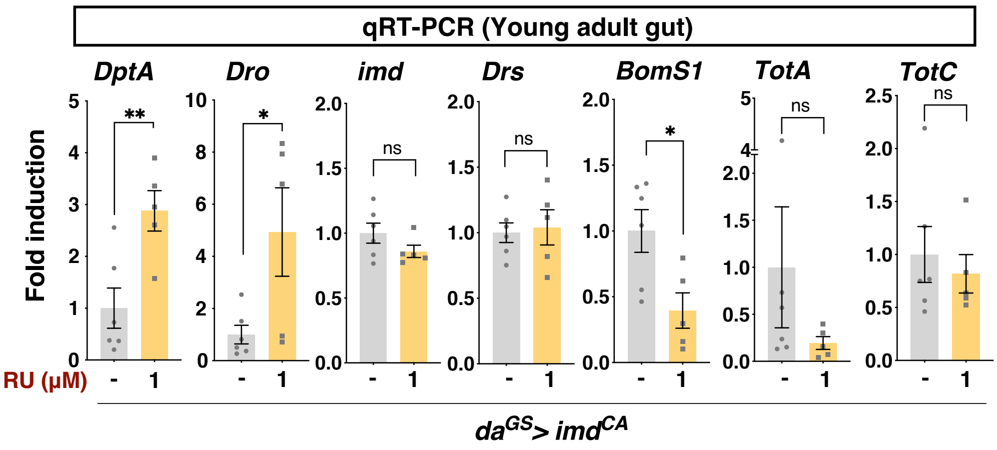

Fig. S5. Quantitative RT-PCR analysis of immune-related genes in young adult gut upon larval Imd activation.

Fold induction of *imd* and its target genes *Diptericin A* (*DptA*) and *Drosocin* (*Dro*), Toll pathway target genes *Drosomycin* (*Drs*) and *Bomanin Short 1* (*BomS1*), or JAK/STAT pathway target genes *Turandot A* (*TotA*) and *Turandot C* (*TotC*) in the gut of day-6-old male flies. n=6 RU- and n=5 RU 1 µM. Each graph shows the mean ± SEM. Statistics: two-tailed Student's *t*-test $*p<0.05$; $**p<0.01$; ns, not significant.

Disease Models & Mechanisms • Supplementary information

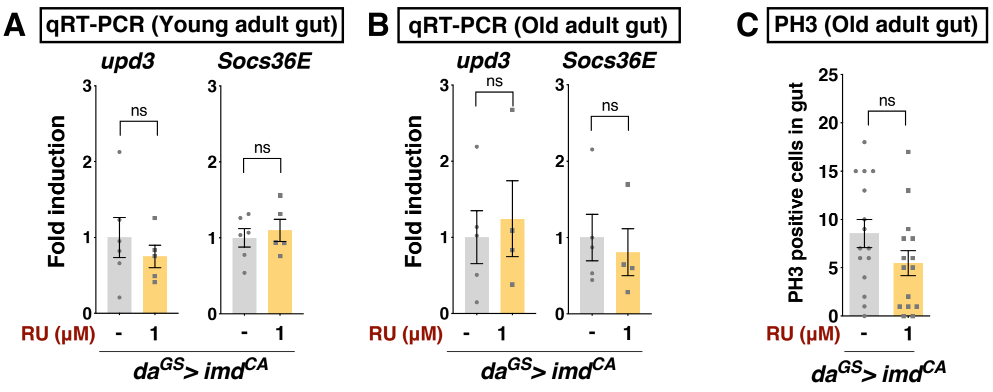

Fig. S6. Larval Imd activation may not induce tissue damage in adult gut. (A,B)**,** Fold induction of upd3 and JAK/STAT target gene *Socs36E* in the gut of young (1-week-old) (A) and old (5-week-old) (B) male flies. n=6 for RU- and n=5 for RU 1 μM. **(C),** Number of phospho-Histone H3 positive proliferating cells in the gut of 5-week-old female flies. n=15. Each graph shows the mean ± SEM. Statistics: two-tailed Student's *t*-test, ns, not significant.

Disease Models & Mechanisms • Supplementary information

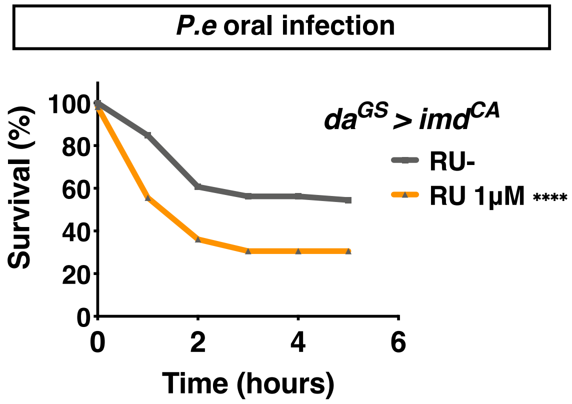

Fig. S7. Adult flies upon larval Imd activation are susceptible to oral infection. Survival curves upon *P. entomophila* oral infection of one-week-old male flies that have experienced larval Imd activation. *daughterless* Gene Switch driver (*da*^{GS}) was used to induce constitutive active form of Imd (*imd*^{CA}) ubiquitously by 1 µM RU486 in the larval stage. n=112 for RU- and n=106 for RU 1 µM. Statistics: log-rank test, ****p<0.0001

Disease Models & Mechanisms • Supplementary information

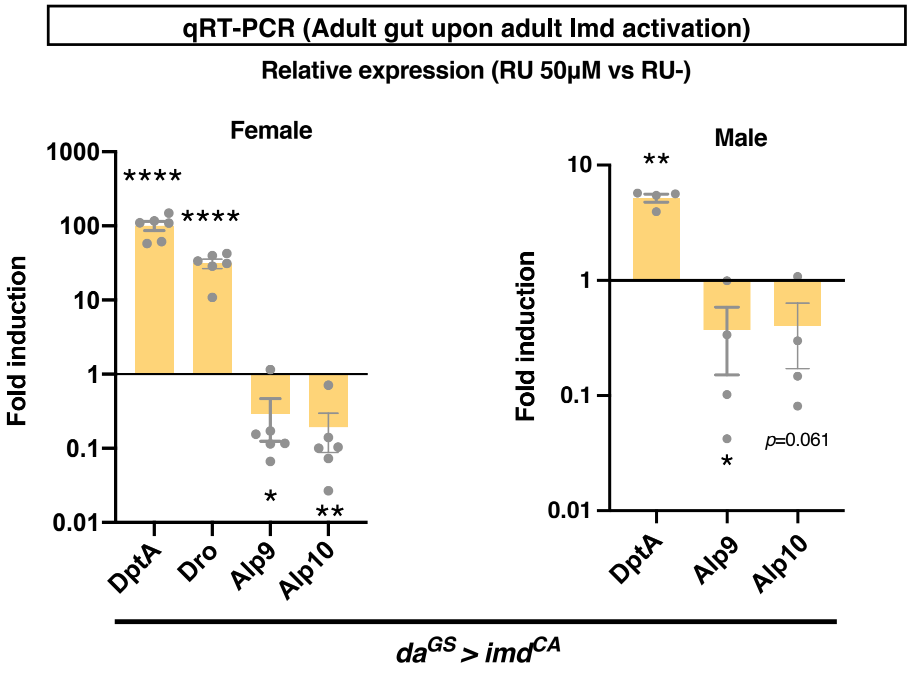

Fig. S8. Imd activation in adult flies decreases IAP expression.

Quantitative RT-PCR analysis of *DptA*, *Dro*, *Alp9*, and *Alp10* in the adult gut. *daughterless* Gene Switch driver (da^{GS}) wad used to induce constitutive active form of Imd (imd^{CA}). da^{GS}>imd^{CA} female and male flies were fed with 50 µM RU486 for 6 days. n=6 for females and n=4 for males. Each graph shows the mean ± SEM. Statistics: two-tailed Student's *t*-test, *p<0.05; **p<0.01; ****p<0.0001; ns, not significant.

Disease Models & Mechanisms • Supplementary information

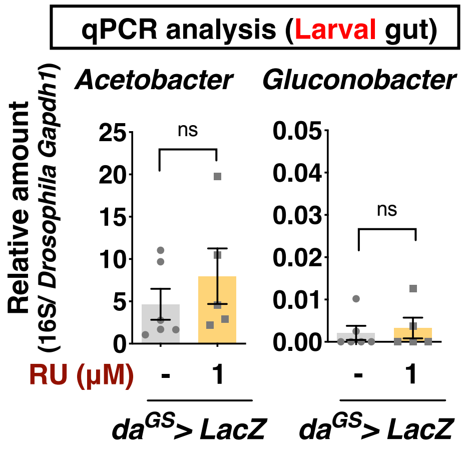

Fig. S9. Absence of the impact on larval gut microbiome by RU486.

Quantitative PCR analysis of *Acetobacter* or *Gluconobacter* in the third-instar larval gut. n=6 for RU- and n=5 for RU 1 μM. Each graph shows the mean ± SEM. Statistics: two-tailed Student's *t*-test, ns, not significant.

Disease Models & Mechanisms • Supplementary information

Table S1. Differentially-expressed genes in the larval gut upon mild Imd activation (Fold change>1.5 or <0.67, adjusted *p*-value<0.05).

Symbol	Gene Name	Fold Change	*p*-value
**Up-regulated genes**			
CecA2	Cecropin A2	61.82	4.91.E-04
CecA1	Cecropin A1	15.21	2.78.E-02
DptA	Diptericin A	13.86	6.27.E-12
DptB	Diptericin B	12.12	1.41.E-03
Zip42C.1	Zinc/iron regulated transporter-related protein 42C.1	11.41	5.30.E-03
AttD	Attacin-D	5.12	2.46.E-06
Dro	Drosocin	5.05	1.69.E-05
edin	elevated during infection	4.76	1.68.E-02
CG14606	uncharacterized protein	3.64	6.30.E-03
CG14205	uncharacterized protein	3.02	1.97.E-03
CG1139	uncharacterized protein	2.73	7.28.E-03
l(2)34Fc	lethal (2) 34Fc	2.50	6.03.E-03
Diedel3	Diedel 3	2.42	3.28.E-03
CG13078	uncharacterized protein	2.38	4.84.E-02
CG32751	uncharacterized protein	2.31	6.98.E-04
CG13325	uncharacterized protein	2.29	1.51.E-04
CG5157	uncharacterized protein	2.26	2.26.E-03
CG15255	uncharacterized protein	2.15	1.51.E-02
Amy-d	Amylase distal	1.97	6.03.E-03
Ugt37A2	UDP-glycosyltransferase family 37 member A2	1.94	5.23.E-03
CG17570	uncharacterized protein	1.91	5.23.E-03
Pdxk	Pyridoxal kinase	1.83	1.41.E-03
CG8773	uncharacterized protein	1.76	1.23.E-02
CG4752	uncharacterized protein	1.70	2.75.E-02
JhI-26	Juvenile hormone-inducible protein 26	1.68	2.75.E-02
Npc2c	Niemann-Pick type C-2c	1.65	2.41.E-02
Alp10	Alkaline phosphatase 10	1.64	3.32.E-02
CG10116	uncharacterized protein	1.57	5.23.E-03
**Down-regulated genes**			
Lcp9	Larval cuticle protein 9	0.089	2.43.E-02
CG17107	uncharacterized protein	0.121	1.74.E-02
Lcp4	Larval cuticle protein 4	0.149	4.91.E-04
CG42500	uncharacterized protein	0.180	4.74.E-03
CG13678	uncharacterized protein	0.212	2.43.E-02
CG10081	uncharacterized protein	0.223	3.98.E-06
Cpr47Eb	Cuticular protein 47Eb	0.321	2.07.E-02
CG11737	uncharacterized protein	0.399	3.72.E-02
CG6277	uncharacterized protein	0.423	8.20.E-04
bmm	brummer	0.508	6.98.E-04
fax	failed axon connections	0.547	2.22.E-03

Table S2. Differentially-expressed genes in the adult gut upon larval Imd activation (Fold change>2 or <0.5, adjusted *p*-value<0.05).

Symbol	Gene Name	Fold Change	*p*-value
**Up-regulated genes**			
Mtk	Metchnikowin	5.95	6.63E-04
DptA	Diptericin A	5.32	3.93E-16
CecC	Cecropin C	4.13	9.41E-07
AttC	Attacin-C	4.10	1.60E-02
Dro	Drosocin	3.80	8.27E-06
CecA2	Cecropin A2	3.55	6.02E-05
CecA1	Cecropin A1	3.45	1.36E-04
lncRNA:CR45045	long non-coding RNA:CR45045	3.38	7.33E-04
Listericin	listericin	2.90	8.38E-03
edin	elevated during infection	2.85	6.79E-03
DptB	Diptericin B	2.72	4.67E-03
AttD	Attacin-D	2.67	4.04E-03
CG4269	uncharacterized protein	2.62	1.42E-02
CG16995	uncharacterized protein	2.59	1.88E-03
Def	Defensin	2.26	3.17E-05
PGRP-SD	Peptidoglycan recognition protein SD	2.23	7.49E-04
CG10383	uncharacterized protein	2.08	1.97E-03
Drsl2	Drosomycin-like 2	2.01	6.67E-04
Cht4	Chitinase 4	2.01	4.87E-02
**Down-regulated genes**			
CG8745	uncharacterized protein	0.303	7.33.E-04
CG7567	uncharacterized protein	0.311	1.29.E-12
CG43673	uncharacterized protein	0.331	1.60.E-03
Alp10	Alkaline phosphatase 10	0.359	1.81.E-04
Alp9	Alkaline phosphatase 9	0.389	4.25.E-02
CG3348	uncharacterized protein	0.397	2.21.E-03
Akh	Adipokinetic hormone	0.416	1.40.E-02
CG32512	uncharacterized protein	0.419	9.15.E-03
CG7231	uncharacterized protein	0.432	1.48.E-02
sug	sugarbabe	0.446	9.94.E-05
CG2680	uncharacterized protein	0.451	7.33.E-04
CG9119	uncharacterized protein	0.461	3.85.E-04
CG33301	uncharacterized protein	0.467	4.03.E-06
CG5346	uncharacterized protein	0.474	3.07.E-07
Ctr1B	Copper transporter 1B	0.475	1.44.E-04
Ser8	Ser8	0.495	2.63.E-02
CG15096	uncharacterized protein	0.496	2.68.E-03
Drat	Death resistor Adh domain containing target	0.496	3.38.E-02

Disease Models & Mechanisms • Supplementary information

Table S3. Primer sequences for quantitative PCR analysis.

Target	Forward primer	Reverse primer
RNA pol2	CCTTCAGGAGTACGGCTATCATCT	CCAGGAAGACCTGAGCATTAATCT
DptA	CGTCGCCTTACTTTGCTGC	CCCTGAAGATTGAGTGGGTACTG
Dro	CCATCGAGGATCACCTGACT	CTTTAGGCGGGCAGAATG
imd	CTCAGTAACGTCCAGACAAACAA	GCCGAAGTGCAGATTATTGGC
Drs	TACTTGTTCGCCCTCTTCG	GTATCTTCCGGACAGGCAGT
BomS1	CACCGTTTTTGTGCTCGGTC	CCCTGCAATCGCCATTGATG
TotA	CCAAAATGAATTCTTCAACTGCT	GAATAGCCCATGCATAGAGGAC
TotC	AATGAATGCCTCCATTTCTCTACTA	GCAACCCTAAGGCTGTCAGA
upd3	ACTGGGAGAACACCTGCAAT	GCCCGTTTGGTTCTGTAGAT
Socs36E	ATGGGTCATCACCTTAGCAAGT	TCCAGGCTGATCGTCTCTACT
Alp9	ATAGCGCCTGTACCTCAACCT	CAGTCGCCTCGCTTCACAT
Alp10	CCAGGCCCGGAGTGATCTA	TTATCGTGCCAGAATCGTGTG
Gapdh1	TAAATTCGACTCGACTCACGGT	CTCC ACCACATACTCGGCTC
Acetobacter	TAGTGGCGGACGGGTGAGTA	AATCAAACGCAGGCTCCTCC
Gluconobacter	CCCAGTGTAGAGGTGAAATTCGT	CCAGGGTATCTAATCCTGTTTGCT

Disease Models & Mechanisms • Supplementary information
